# VR-assisted aggression treatment in forensic psychiatry: a qualitative study in patients with severe mental disorders

**DOI:** 10.3389/fpsyt.2024.1307633

**Published:** 2024-05-16

**Authors:** Fernando Renee González Moraga, Pia Enebrink, Sean Perrin, Kristina Sygel, Wim Veling, Märta Wallinius

**Affiliations:** ^1^ Evidence-based Forensic Psychiatry, Department of Clinical Sciences Lund, Psychiatry, Lund University, Lund, Sweden; ^2^ Research Department, Regional Forensic Psychiatric Clinic, Växjö, Sweden; ^3^ Centre for Ethics, Law and Mental Health, Institute of Neuroscience and Physiology, the Sahlgrenska Academy at the University of Gothenburg, Gothenburg, Sweden; ^4^ Division of Psychology, Department of Clinical Neuroscience, Karolinska Institutet, Stockholm, Sweden; ^5^ Department of Psychology, Lund University, Lund, Sweden; ^6^ Department of Forensic Psychiatry, National Board of Forensic Medicine, Stockholm, Sweden; ^7^ Department of Psychiatry, University Medical Center Groningen, University of Groningen, Groningen, Netherlands

**Keywords:** virtual reality, VR, aggression, forensic psychiatry, VRAPT, experiences, treatment, content analysis

## Abstract

**Introduction:**

Improvements in virtual reality (VR) have made it possible to create realistic, virtual settings for behavioral assessment and skills training that cannot otherwise be accessed in a safe way in forensic psychiatric settings. VR interventions are under development but little is known how forensic psychiatric patients with severe mental disorders experience VR-assisted assessments or treatments.

**Methods:**

The present study aimed to help fill this knowledge gap via qualitative interviews with seven patients with severe mental disorders at a high-security forensic psychiatric clinic who had completed the newly revised Virtual Reality Aggression Prevention Training (VRAPT). All participants were interviewed 12 weeks after the VRAPT intervention, and interview data analyzed with manifest inductive content analysis.

**Results:**

Six manifest content categories were identified: 1. Therapeutic process, 2. VRAPT method, 3. VR technology, 4. Previous treatment experiences, 5. Challenges to treatment of aggression, and 6. Unexpected experiences. The participants had diverse experiences related to both the VRAPT intervention and forensic psychiatric care. Participants described a mixture of positive experiences in relation to VR-assisted role-plays, and less positive in relation to motivation for aggression-focused treatment and technological limitations.

**Discussion:**

The present findings suggest further studies are needed on how to best implement VR-assisted treatments for aggression in forensic settings, and potentially further modification of treatment content in interventions like VRAPT.

## 1 Introduction

Forensic psychiatric patients have been described as a heterogeneous, challenging, and vulnerable group in both society and clinical settings (1–3); their behaviors and clinical status can be traced to a complex constellation of severe mental disorders, antisocial lifestyle, substance use, and a high degree of impulsivity, and/or lack of empathy (4–6). Patient aggression is a known problem in forensic psychiatry and is considered central to patient management (7, 8), yet there is a scarcity of evidence-based treatments for aggression. There is a strong need for more research to develop and evaluate aggression treatments with all the challenges this implies (9–14). Recently, immersive virtual reality (VR) has been identified as a potential tool in assessment and treatment interventions in forensic settings (14–16), also with possibilities for treatment of aggression (9).

Three core concepts in VR have been described: immersion, presence and embodiment (17–20). VR is an immersive technology in that immersion can be regarded as a quality of the system; the system presents a vivid virtual environment while shutting out physical reality (21). Thus, VR technologies can be more or less immersive depending on the quality of the system used (22). Presence, on the other hand, is a perceptual illusion of *being there*; this is not only a cognitive illusion since your body reacts to the illusion even though you are aware that you are not there – your brain cannot overcome the illusion (20). When VR technology works as intended, the user accepts, interacts and is physically, socially and emotionally engaged in the virtual world (23, 24). Embodiment, or sense of embodiment in VR, is how we incorporate and experience our body in a virtual body in VR and is an important factor to the sense of being in a virtual environment (25). Three components have been associated with embodiment: sense of self-location, sense of agency, and sense of body ownership (26).

In forensic settings, VR holds the potential to increase motivation among patients, bridge the gap between real life, therapeutic and laboratory experiences, and increase ecological validity of psychological research (27–31). Understanding the individual’s motivation for the aggression (32) and the function of their aggression [e.g., social recognition, emotion regulation, communication, protection; (33)] are important components in comprehending why aggression has emerged and how to treat it, and thus these concepts play an important but understudied role in the development of VR treatments for aggression. Different ways that motivation and function of aggression have been assessed is from first perspectives among youths [see Angry Aggression Scale, AAS; (34)], based on the quadripartite violence typology [QVT: (35), psychopathy (36)], and through staffs’ perspectives of forensic patients’ aggression (see Assessment and Classification of Function, ACF; 37). An integrated review (38) found that psychiatric inpatient’s perceived aggression and violence were associated with a lack of respect by staff towards patients, coercive or controlling interventions in their care, poor staff interpersonal skills, boredom/lack of structured activity, lack of privacy/personal space in the care environment, medication issues and patients’ personal characteristics.

Today, two methods employing VR in treatment of aggression in forensic settings are available: VR Game for Aggressive Impulse Management [VR-GAIME; (39)] and Virtual Reality Aggression Prevention Training [VRAPT; (40)]. Both methods were primarily developed for forensic psychiatric patients. In the first randomized controlled trial of VR-GAIME with 30 forensic psychiatric outpatients, VR-GAIME was not more successful in reducing anger and aggressive behavior relative to the control condition, but demonstrated ways in which future work may realize the unfulfilled potential of combining serious gaming and VR in creating effective aggression management interventions (41). The first randomized controlled trial on VRAPT with 128 forensic psychiatric patients with aggression problems, found positive effects at post-treatment as indexed by self-reported hostility, anger control and non-planning impulsiveness, but these effects were not maintained at follow-up (42). While VR-assisted aggression interventions applicable to forensic settings hold the promise of safer and more ecologically valid treatments, the current evidence base is insufficient to draw conclusions about their acceptability and efficacy (42–44), underlining the urgency for more research in this area (9, 31).

**Figure 1:**
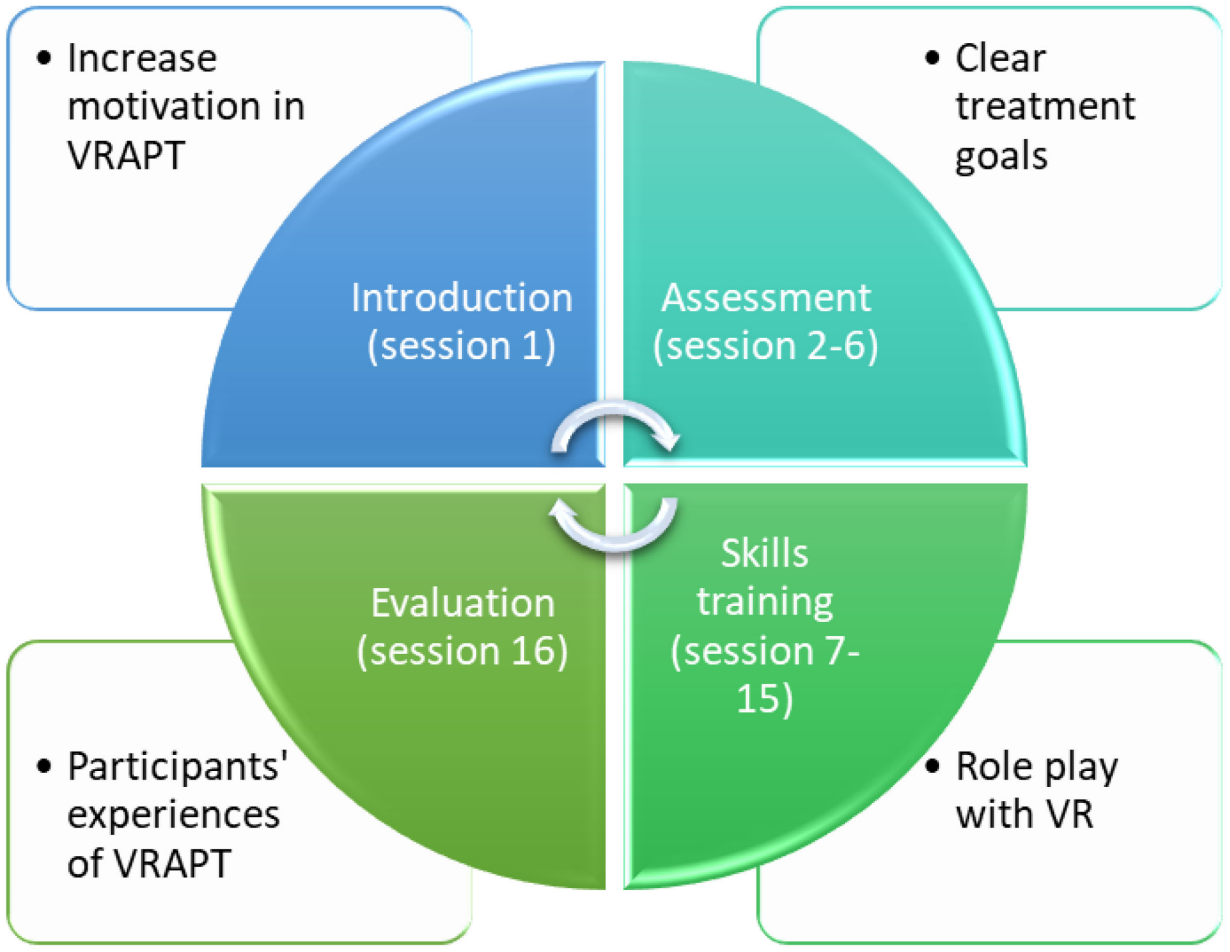
Structure of Virtual Reality Aggression Prevention Treatment (VRAPT).

Patients’ experiences of their treatments are important to our understanding of the responsiveness of these treatments (to the patient) which will in turn influence motivation, treatment engagement and efficacy (14, 45–48), providing directions for further treatment developments. Recently, the VRAPT intervention was revised (9) and pilot studies carried out in forensic psychiatric and prison settings (49). In its revised version [see (9)], VRAPT aims to increase the participant’s understanding and management of his/her dysfunctional and reactive aggressive behaviors through a CBT approach theoretically underpinned by the General Aggression Model GAM (50–52). During the initial assessment phase (see **Figure 1** for an overview of VRAPT treatment phases), a historical and functional analysis of the participant’s dysfunctional aggressive behavior is made in collaboration between participant and therapist. This is then used as a guide for all continued skills training during the skills training phase. For example, a participant may practice situations that are difficult to cope with on a daily basis and that previously have caused the participant to react aggressively and/or even commit a crime because he/she has not known how to manage the situation without resorting to aggression. For instance, the therapist can simulate a stressful situation for the participant through information obtained from the participant regarding previous experiences. Examples of environments that can be simulated with the software Social Worlds ^©^CleVR are a park, a bus, a café, a jail, a meeting room, and a home environment. In the virtual environment, the therapist can tailor the simulated scenario in real time, e.g. facial expressions, voices, verbal communication and movements of the avatars, while simultaneously seeing what the patient is experiencing in the environment.

The current study constitutes a qualitative investigation of forensic psychiatric patients’ experiences of undergoing the revised VRAPT treatment with the following research question: How do patients experience VRAPT and its consequences, opportunities and challenges in a high-security forensic psychiatric clinic?

## 2 Materials and methods

This is a qualitative study applying inductive content analysis (53, 54) on data from interviews with forensic psychiatric patients who were interviewed 12 weeks after completion of the VRAPT intervention, at the time of a quantitative follow-up of the treatment, between February and October 2021. The colored portion of **Figure 1** below gives an overview of the structure of VRAPT [see also **Table 1** in (9) for further details of the treatment], note that the proportion of sessions for each phase is not equal, with most sessions considering skills training.

**Table 1:** Content categories and subcategories of forensic psychiatric patients’ experiences from the VRAPT intervention.

Category	1. Therapeutic process	2. VRAPT method	3. VR technology	4. Previous treatment experiences	5. Challenges to treatment of aggression	6. Unexpected experiences
**Subcategory**	1.1 Therapeutic alliance	2.1 Treatment outcomes	3.1 Novelty	4.1 Previous experiences from aggression treatment	5.1 Manipulation	6.1 Covid-19
	1.2 Treatment goals	2.2 Homework experiences	3.2 Immersion	4.2 Patients’ experiences of forensic psychiatric care	5.2 Positive experiences from aggression	
	1.3 Matching of treatment to patient’s needs	2.3 Staff involvement	3.3 Physiological side effects			
	1.4 Skills training in treatment	2.4 Deviations from VRAPT methodology	3.4 Limitations of technology on therapeutic work			
	1.5 Remaining treatment needs	2.5 Suggestions for improvement	3.5 Potential of VR in forensic settings			

### 2.1 Ethical considerations

This study was approved by the Swedish Ethical Review Authority (Dnr: 2019-02337; 2020-06317). Eligibility for participation was first assessed by the patients’ treating psychiatrist, taking into account their need for aggression treatment, capacity to make an informed decision on participation (e.g., not being under the influence of acute psychosis or intellectual disability), and the possibilities to participate safely in relation to imminent risk of severe violence. Written informed consent was obtained from all participants after providing them with a verbal and written explanation of the study’s purpose and procedures. Participation was voluntary, and participants were told that they could withdraw from the study at any time without having to explain why they decided to do so and without impacting any other aspects of their ongoing care. Participants received a ticket for 99 SEK (~€9) to use at the clinic kiosk after the study was completed. All data were pseudonymized before analysis and recordings deleted after the analyses were finished.

Taking into account the persuasiveness of VR technology (55) and the intrinsically coercive nature of forensic settings (56), some studies suggest that ethical priorities must account for the specific vulnerabilities (e.g., autonomy, deception, informed consent, mental liberty, moral agency, dignity) of (forensic) psychiatric patients when using new therapeutic technologies with this population (56–58). For example, therapeutic misconception (9, 59) refers to the fact that a participant may not fully differentiate between participation in clinical research and an ordinary treatment. In this study, we tried to minimize the risk for therapeutic misconception through thorough information to the participants regarding the distinction between the study and their ordinary care. However, there were occasions when participants expressed wishes that the results from their participation in the VRAPT research could be reported to the psychiatrist responsible for their treatment, as a means to affect their care process. All such requests were denied, with reference to the information they received prior to participation.

### 2.2 Participants

Participants were seven forensic psychiatric patients (6 males, 1 female; mean age = 36 years old; range = 22 – 46 years old), recruited from a high-security forensic psychiatric clinic in Sweden. These seven participants constituted all forensic psychiatric patients who completed the full VRAPT treatment within a quantitative pilot study. All participants had been assessed with a severe mental disorder during a forensic psychiatric investigation before being sentenced to forensic psychiatric care. Inclusion criteria were: 1) ongoing treatment under the law of forensic psychiatric care, 2) history of aggression and current problems with reactive aggression, and 3) having undergone VRAPT treatment. Exclusion criteria were: 1) inability to speak and read Swedish, 2) epilepsy, 3) intellectual disability (IQ < 70), 4) and/or severe autism spectrum disorder, 5) acute psychotic state, and 6) security risks that prohibit participation. The current psychiatric diagnoses (collected from participants’ medical files) at the time of participation were schizophrenia spectrum disorders and antisocial personality disorder (both n = 4; 57%), substance use disorders (n = 3; 43%), personality disorder – trait specified (n = 2; 29%) and borderline intellectual functioning, autism, bipolar disorder and paraphilia (all n = 1; 14%).

This study was conducted as part of a larger study, with an accompanying quantitative VRAPT evaluation. All participants (N = 7) who completed the VRAPT intervention as part of the quantitative evaluation, were invited to participation in this study, of which all accepted (100% participation rate). In qualitative research, data saturation must be considered, with suggested guidelines of 9-12 interviews for data saturation (60). In this study, with a narrow focus and a specific group, we had a total sample on N = 7. However, given the data available, our consensus was that data saturation was achieved with the current sample size.

### 2.3 Procedure

All interviews were conducted by the main author (FGM), a licensed clinical psychologist with training in qualitative methodology and experience working in high-security forensic psychiatry. The interviews were semi-structured and based on an interview guide developed by two of the authors (FGM, MW) for this study. The interview contained open-ended questions such as “How did VRAPT contribute to your own management of aggression?”, “How did you experience using VRAPT as a treatment intervention”, with the opportunity for follow-up questions based on the participant’s previous answers. All interviews were audio-recorded and transcribed for analysis purposes. Interviews were conducted in the clinic on a one-to-one basis except for one case where a staff member was present during the interview due to security reasons. The interviews varied in length from 13 to 34 minutes.

### 2.4 Analyses

This study applies an inductive approach to qualitative manifest content analysis (53) to examine patients’ experiences of the VRAPT intervention. In particular, we considered recommendations from content analysis for data organization and analysis (61–63). After transcription, the interviews and transcriptions were listened to and read several times to correct the transcription and facilitate the overall comprehension of the data. Thereafter, preliminary codes were assigned. After codes were assigned, data were ordered in content categories and subcategories. A content category describes the content on a manifest level, with a low degree of interpretation and a varying degree of abstraction (63). FGM performed the initial coding, then FGM and MW independently identified content categories and subcategories. Final categories were refined and determined in consensus between FGM and MW. Thereafter, categories were interpreted and a narrative summary of the main findings determined. No software was used in the analysis process.

## 3 Results

During the interviews, the following content categories emerged: 1. Therapeutic process, 2. VRAPT method, 3. VR technology, 4. Previous treatment experiences, 5. Challenges to treatment of aggression, and 6. Unexpected experiences. **Table 1** provides an overview of content categories and subcategories of forensic psychiatric patients’ experiences from the VRAPT intervention. Each of the categories and subcategories are described below.

### 3.1 Therapeutic process

This content category included participants’ feelings and thoughts associated with the therapeutic process during the VRAPT intervention. There was a wide range of participants’ experiences, with some comments being very specific to the participant’s own experiences from the VRAPT intervention, and some comments about the general treatment experience, both VRAPT and the treatment milieu on the care unit. The following subcategories were identified: Therapeutic alliance, Treatment goals, Matching of treatment to patient’s needs, Skills training in treatment, and Remaining treatment needs.

#### 3.1.1 Therapeutic alliance

During interviews, participants described how they experienced the treatment relationship to the VRAPT therapist. Several participants perceived the relationship with their therapists in a positive way, highlighting the collaboration and emphasizing the importance of feeling safe during the session. Preparing the participant for what was to come during the session seemed to facilitate a feeling of safety for the patient.

*“My therapist had, I think, a genuine sense of developing the patient in the flaws that they possess.” (P4)*

*I thought we had a good collaboration, he was clear and told me each time what was going to happen and so on. So he was good, I think … I mean, he told me exactly what was going to happen, so I was clear beforehand, I could feel safe. Then you never know how you will react.” (P7)*

Another participant described the treatment relationship with the therapist as difficult and that feelings of sadness and stigmatization were triggered.

*“(My therapist) tried to oppress me, I felt sad and bad but still thought oh I’ll manage this and did it … I felt humiliated and sad, didn’t want to talk to anyone, just wanted to be left alone. Why did (my therapist) do that? I am also a human, I have made a mistake and am here to pay for it, doing all the programs, treatments, attending meetings and there is nothing more. I am also human, also have feelings, think about that. Just like everyone else.” (P5)*

#### 3.1.2 Treatment goals

During the first VRAPT sessions, the participant and therapist formulate treatment goals. In some interviews, the participants had ambiguous, or negative, experiences concerning treatment goals.

*“I cannot really answer that because no one said that we would have a goal, we just started there and so … I would probably say that it’s about remembering them and trying to move forward. Putting energy into thinking about things, implementing and feeling that you know what direction to take … Trying to deal with difficult situations in a new way…” (P2)*

*“If we had a goal? No we didn’t have a goal.” (P3)*

For other participants, treatment goals were described as an important part of the treatment, incorporating previous experiences and giving the participant directions for the continued development.

*“My goals were to be able to de-escalate challenging situations and currently I don’t remember quite how it was formulated, and I practiced that a lot, based on previous experiences I had and got during the time in VRAPT, you went back to those experiences and implemented in the goals so it would go together with the goals you had … The goals kind of gave directions you should take, and it was just to follow the road.” (P4)*

#### 3.1.3 Matching of treatment to patient’s needs

A crucial component in all therapeutic interventions is matching treatment to the participant’s needs. VRAPT is a new intervention, not familiar to participants or therapists in general. During interviews, descriptions by participants that VRAPT was not what they initially expected, or that the intervention did not match their own perception of treatment needs, were discerned.

*“… I began to realize more and more that this thing with the VR study was about me doing a treatment to become less aggressive, not that I was just going to go through these things and the method. We then had some rather long conversations, several times in a row, where we concluded that I am not interested in being treated for aggression. It demanded more than I can handle.” (P1)*

One participant referred to previous treatment experiences and how VRAPT failed to provide new strategies to manage difficult situations.

*“Then it was this that you did not learn so many strategies, it was mostly breathing and such, was mostly that you went through the situation but there was not much you learned on how to handle the situation but they came during VRAPT and then you acted according to the situation but there was not so much that I learned new strategies. This about breathing and things, I have already learned that from (my previous therapist).” (P2)*

#### 3.1.4 Skills training in treatment

The overall aim of VRAPT is to treat aggression through increased awareness and skills training. Skills training is performed in VR-assisted role-plays with participants, intended to increase participants’ awareness of their own reactions, and management of reactions, during conflict resolution. This is important for treatment outcomes since participants’ past dysregulated reactions have been maladaptive, generating problems for themselves and others. These skills training sessions may be challenging to participants and yet are a crucial part of the intervention. Initial unease related to skills training could, for some participants, be replaced with a sense of understanding, as continued skills training could provide insights into their own strengths and weaknesses, specifically in social situations.

*“Very difficult, you put yourself in situations where you are challenged … Yes, it is still a bit difficult. But it will always be, to be honest. Yet it is fun at the same time because you are stimulated to be a stronger person even though it is difficult…” (P4)*

*“In the beginning it was a bit unclear, then afterwards it just became understandable that you can use this to give rise to certain feelings or thoughts … you get used to it … It changes, it’s just like with everything else in the beginning when you do things for the first time it usually feels a bit different but the more you practice, put time into it the easier it feels … The more you got into the VRAPT and the project, you got into how you are as a person. What your weaknesses are and what your strengths are in a social context…” (P4)*

*“I thought it was okay, but it can be a bit difficult when the person I’m arguing with is arguing against me, when there’s not much to say. There were certain situations where I tried to reason with the person, but it didn’t go well…” (P5)*

#### 3.1.5 Remaining treatment needs

When asked about remaining treatment needs after the completion of VRAPT, participants specifically mentioned different kinds of skills training, especially concerning communication and managing difficult situations in which you feel challenged or experience severe anger. Some participants had experienced difficulties with emotion regulation post VRAPT treatment and emphasized how they wished they had been able to access their learned skills to avoid ending up in unfavorable circumstances.

*“I would like to work more on situations where you feel challenged as a person.” (P4)*

*“It would have been to communicate even more … Yes, to become even better at it and to stop in time. So I don’t get so damn angry … I should have thought more about what the therapist said so I wouldn’t have ended up in this situation I’m in now. I got so damn angry again. I had forgotten everything I had learned and thought it was tragic as hell.” (P7)*

### 3.2 VRAPT method

Part of the interviews concerned the specific VRAPT intervention, with the following subcategories: Treatment outcomes, Suggestions for improvement, Homework experiences, Staff involvement, and Deviations from VRAPT methodology.

#### 3.2.1 Treatment outcomes

The subjectively experienced outcomes from the treatment varied significantly between participants. For some, participation in the VRAPT intervention did not yield specific effects regarding skills training in management of aggression due to absence of aggressive impulses to manage during the sessions. Yet, an ambivalence in relation to perceived outcomes was clear in the data, where addressing aggression and learning new strategies for interpersonal interactions were described as beneficial by participants.

*“Well, the thing is that I didn’t learn anything about how to reduce aggression because I didn’t get any. So the technique failed quite early on … But there was a result because we talked about aggression…” (P1)*

*“There were both situations where I could recognize myself and those that were new, but I learned the new ones pretty well … I don’t know, I didn’t think it was very useful … Yes, the only thing I thought changed was that I used the strategy of being diplomatic instead of going out and punching someone … It did help, I noticed that when I tried to be calmer, the situation became easier than if you started arguing. It may help if there’s a situation on the ward where a patient is arguing a lot, of course it’s positive to be able to take it easy instead of getting involved.” (P2)*

The opportunity to learn new communication and interaction skills and manage emotions in difficult situations was highlighted by several participants. For some, practicing emotion recognition in others was described as beneficial.

*“Different strategies like counting slowly and breathing calmly. Have learned quite a lot actually … The advantage is that you learn to recognize facial expressions and the negative is that there are many hits you have to make.” (P3)*

*“The care has changed for me after VRAPT. I am more alert and awake to the situations I face … My own emotions are more stable … I used to find it easy to deal with anger but now it’s easier. I have learned more about myself. I have learned what my weaknesses are…” (P4)*

*“I learned to recognize and interpret facial expressions. Then I also learned to be able to handle stressful situations in a calm way … Then I learned to handle situations in a good way … There were situations that were stressful, for example when a security guard or police officer accused me of having done something, then I tried to reason and handle the situation…” (P6)*

Managing situations which give rise to tension and irritation in a constructive way while at the same time considering your own needs, was described as important. In this regard, practical skills training seemed to be crucial for the participants.

*“What I learned the most from was when you had to relive the situation where you felt tense, irritated and angry. The role play, that’s where I learned the most … Because it was a different way to digest different situations that you experienced. Normally you sit and think and reflect on things that have happened, but here you kind of got to experience it in a completely different way and also act at the same time … The most challenging thing for me was to talk about how to respect yourself as a person when dealing with other people. I was good at arguing against and respecting the other person, but respecting myself was lacking. To kind of tell how I feel in the given situation…” (P4)*

*“It gave me many tools on how to act in many situations. I thought that … Yes, you should try to think about communicating, that’s the only thing that solves everything, no violence … I learned more about stopping in time and so on … It was probably mostly when I had to communicate that I learned the most.” (P7)*

#### 3.2.2 Homework experiences

Several participants shared negative experiences of the homework assignments, some more related to repetitive aspects of the homework, and some due to associations to previous school experiences.

*“I didn’t think it was good because there were a lot of the same questions all the time. It became annoying that it didn´t differ.” (P2)*

*“It was like going to school again. You had to write homework and things like that.” (P3)*

However, some participants described the homework assignments as a point of orientation and skills training during VRAPT, and that help from ward staff to remember the assignments was important.

*“It gave structure to the whole project, that you had homework such as, okay, this week you should write down which situations you have experienced, how they have affected you, what thoughts and feelings have emerged, it became more structured to use it … It has been fun and rewarding, then you have to think, you had to do that…” (P4)*

*“They were good but the hard part was remembering to do them. (*If the participant needed support*) Yes, I asked the staff but they didn’t always remember, so there were some times when I didn’t do the homework…” (P6)*

#### 3.2.3 Staff involvement

The participants undergoing the VRAPT intervention were all within an inpatient care setting, with 24/7 staff availability. Thus, there were possibilities for ward staff to be involved in the intervention, for instance with homework assignments. Yet, ward staff involvement seemed to have been minimal. In some cases, this was preferable from the participant’s perspective, but in other cases this was described as a shortcoming of VRAPT.

*“Other staff have not been so involved in VRAPT, but they more asked “what are you doing now?” and if you explained that you are doing your homework, they saw you sitting with the notebook and doing your homework. They talked a bit about it but not more than that.” (P4)*

#### 3.2.4 Deviations from VRAPT methodology

VRAPT is a manualized treatment, with a manual complemented by therapist and participant workbooks to guide the treatment. Tailoring of the treatment content to participants’ needs is possible, but the general concept should be followed for the intervention to be identified as VRAPT. During the interviews, examples of care interventions that were not part of VRAPT methodology yet took place during the intervention, were described. In some cases, this may have been related to general treatment needs in the patient — a need for someone to talk to about the symptomatology associated with psychiatric illness. In other cases, skills training according to the manual was performed but outside of VR.

*“Then we talked a lot, we talked a lot about things that had nothing to do with this VR study.” (P1)*

*“So what me and my supervisor did was that we role-played outside VR, face to face.” (P4)*

#### 3.2.5 Suggestions for improvement

During the interviews, participants were asked to describe what they believed needs to be improved in the VRAPT intervention. More time for skills training in VR was a common wish, both more time during the individual sessions to be able to penetrate the participant’s management skills, but also possibilities for an extension, or booster sessions, of VRAPT.

*“That you should have gone through the situation a little more than you did and how you reacted and so on … We had an hour but actually it would probably have taken longer than an hour for each session … felt that I would have needed a little more time each time.” (P2)*

*“…I would like to go to a continuation level or whatever you call it. Then I would have liked to be involved, but I guess that doesn´t exist…* (About wanting to have a continuation level) *Yes, three or four more months.” (P7)*

Other specific parts that were suggested for improvements were the workbooks but also parts related to the VR technology, e.g. facial expressions of the avatars.

(About the workbooks) *“It should have been rephrased a bit and been different, it would have been easier and more interesting, if you had varied the questions a bit more…” (P2)*

(about emotion recognition) *“Yes, I wish they had improved that because they are so similar. Wait, what was it now, one was angry, one looked disgusted and those two were very similar and it is very difficult to choose which is which…” (P7)*

### 3.3 VR technology

Another content category evident in the data was content related to the VR technology per se. All VRAPT sessions except for the last session included time in the virtual world. That is, the participants in this study had several hours of experience in the VR environment, and the following subcategories of participants’ experiences were discerned in the data: Novelty, Immersion, Physiological side-effects, and Limitations of technology on therapeutic work.

#### 3.3.1 Novelty

For most participants, the use of VR was novel, which increased their interest in participating in the intervention. At the beginning, this involved many new experiences and could be both thrilling and uncomfortable to a certain degree. However, the majority of the participants shared positive experiences of using VR technology.

*“My experience over time was that I thought the first times I tried it, it was fun because it was new and so on.” (P1)*

*“At first it felt a bit strange to use those glasses, like entering a completely different world. I’m not used to video games or anything like that. It felt a bit strange but at the same time exciting … It felt good. It was fun…” (P7)*

#### 3.3.2 Immersion

A central concept in VR research and practice is immersion. The participants described how they experienced taking part in the VR experience, where some described a lack of immersion.

*“That it would feel like you were in another world and lost everything around you but I didn’t really get that experience…” (P1)*

*“Then you would forget that it’s the VR world, but you still did that to some extent … The advantage is that you are challenged to become stronger and the disadvantage is that it is virtual. By that I mean the role-plays, as I said, you couldn’t immerse yourself 100%.”* (P4)

When asked specifically about these experiences, participants referred to technological limitations in graphics and sound including voice distortion.

*“It was bad graphics, the graphics engine itself was bad, for instance you couldn’t sit down anywhere, it was very limited … No, it was just the psychologist’s voice, but it was mixed and I was always aware of it … I was always aware that this with VR had a purpose, it became artificial in some way … It’s too unrealistic.” (P1)*

*“In VR, you are like a robot…(Interviewer: If I understand you correctly, the voices made you lose the feeling of being there, like you disconnected from it at certain moment) Yes, sometimes I even started laughing because it feels so unreal. But you tried to focus on the task.” (P4)*

For other patients, VR provided a possibility of being emotionally connected to the virtual world and, to a certain point, VR glasses became an almost non-existent accessory.

*“It was emotional.” (P5)*

*“It’s quite interesting, it feels like you end up in a different world once you put the glasses on, the headphones … It almost felt like the real world, except that it was simulated and maybe not as good graphics, but it felt very real … It can be heavy with the equipment but you forget about it once you are in the VR world, or you notice afterwards that it has been quite heavy. You don’t notice it at the time…” (P6)*

#### 3.3.3 Physiological side-effects

Some participants reported physiological symptoms when using the VR equipment, symptoms which gradually diminished with the passing of the sessions as they became accustomed to using VR.

*“When you put on the VR glasses and have the controls in your hands and have to learn on-site, you might feel a bit dizzy, not really dizzy but you lose your balance a bit … It happened the first times but then you get used to it…” (P4)*

#### 3.3.4 Limitations of technology on therapeutic work

During interviews, some of the previously described limitations and lack of outcome for participants were related to limitations of the technology used in the current study. This concerned both the VR technology and the wristwatch used to measure skin conductance and heart-rate variability. This affected not only the participant’s experience during the session but also the participant’s general view on participating in a treatment evaluation. Situations where the technology did not correspond to what is possible in reality was described as a limitation.

*“…a lot of the equipment didn’t work. For example, we should measure heart rate and sweating. It happened that I had a pulse of 30 and sometimes it was up to 250, it didn’t work, something was wrong. Thus I became a bit skeptical about the study itself…” (P1)*

*“…but you would also have been able to just walk away because it’s also a strategy to just walk away and you didn’t have that in those situations and I think that’s a bit stupid.” (P2)*

#### 3.3.5 Potential of VR in forensic settings

When participants were asked about the possibilities of VR in forensic settings, different kinds of skills training were highlighted.

*“Improving for patients. It might be … aggression and practicing interacting with others, practicing everyday life, going to the bank and everything possible.” (P6)*

### 3.4 Previous treatment experiences

While the interview guide was specifically focused on VRAPT and VR, content related to participants’ previous treatment experiences, both considering specific treatments but also concerning general treatment in forensic psychiatry, emerged. This was conceptualized in the subcategories: Previous experiences from aggression treatment and Patients’ experiences of forensic psychiatric care.

#### 3.4.1 Previous experiences from aggression treatment

Despite several of the participants having had experiences of different kinds of psychological treatments, none of them could describe ever having received any aggression-specific treatment. In some cases, the participants even shared experiences where therapists had refused to talk about aggression and that psychotherapy was aborted due to lack of progress. VRAPT was described as something novel, with possibilities for deeper penetration of participant’s needs.

*“Yes, I did have a few conversations with (my therapist), it’s actually the first time I’ve gone through it with a psychologist, it never happened at the other clinic, no psychologist brought it up, they just said no…*(if the care provider talked about aggression) *No, it went so far that (previous therapist) terminated the contact with me, she couldn’t understand me. Now the doctor has to take over with medication, she said, seven or eight years we had sat and talked and then she quit.”* (P1)

*“I haven’t experienced so many. I’ve had psychological sessions and that’s the only thing I can compare it to, and I’d like to say that VRAPT is more in-depth than regular sessions with a psychologist…” (P4)*

#### 3.4.2 Patients’ experiences of forensic psychiatric care

During the interviews, some participants reflected on experiences of forensic psychiatry in general, sharing negative memories and experiences of missing out on things happening in the outside world. Others described how being locked up affects them and may give rise to feelings of irritation.

*“You know, I have been institutionalized since (several years) when I was incarcerated. It’s hard. It’s ward after ward, a corridor with an area of one meter where 15 people live … My own feelings are what I’ve gone through meeting after meeting for (several years) years, it takes a bit of a toll on my psyche but what can I do … I’m bitter, I’m done and ready to be treated in outpatient care. It feels like I’ve been here too long, the crimes were a long time ago but I haven’t shown any day that I’m angry.. I also have feelings, mom, dad, siblings … I have lost my (relatives). I haven’t been able to attend any funerals.” (P5)*

*“Being imprisoned can make me irritable, and rules and restrictions.” (P6)*

### 3.5 Challenges to treatment of aggression

VRAPT was conceived as a treatment directed towards reactive aggression. In the interviews, it could be observed that aggression was more complex than that in this sample of forensic psychiatric patients. For some, instrumental aggression was the predominant form of aggression. For others, aggression stemmed from a severe mental illness, such as psychosis. Further complicating things, different types of aggression may have been present at different points in participants’ lives. In the data, descriptions where participants had specific aims with their actions during therapy, or in relation to aggressive acts, that could pose significant challenges to treatment of aggression were visible. Two subcategories were discerned: Manipulation and Positive experiences from aggression.

#### 3.5.1 Manipulation

Some participants were very aware of the therapist’s intentions during treatment, so they just “played the game”.

*“You also noticed that some situations were made from the outside where the therapist thought what I was triggered by, so I did the opposite because I understood what (the therapist) was looking for. That (the therapist) wanted a reaction or something…” (P2)*

#### 3.5.2 Positive experiences from aggression

For other participants, aggression was perceived as something positive, and fantasies of harming others were common and fulfilling for the participant.

*“Especially now after this, I had assured everyone that I would not do anything, but still it happened. Almost 10 last minutes without my own will. So for me, the aggression is not just negative, I get a positive experience from it … I’ve fantasized about hurting people a lot on the ward, but it’s only been in the realm of fantasy then.” (P1)*

In some cases, the desire to hurt others was described as so intense that it was unthinkable to undergo an aggression treatment. Also, previous experiences of failed treatments paved way for a negative view on ever being able to succeed with aggression treatment.

*“…I do find so many positive things in having these violent fantasies, I don’t want to get rid of them … I personally don’t suffer much from having this problem. I feel an enormous unwillingness to do anything about it because I’ve been in so much therapy that I’ve come to the conclusion that I’ll never get rid of this, I feel hopeless when I think about dealing with the situation in a better way … I’ve almost given up on the idea that I could prevent myself from committing violent crimes…” (P1)*

### 3.6 Unexpected experiences

#### 3.6.1 Covid 19

During the course of a study, it is not uncommon for unexpected events to occur that may affect the research. In this case, as therapists began to see the participants in the VRAPT intervention, the first cases of Covid-19 appeared in Sweden. This led to a number of actions being taken to reduce patient-therapist contact, as well as actions to reduce the likelihood of patients infecting each other and, most of all, to prevent staff from entering the clinic with the virus. Given these circumstances, a specific part of the interview concerned how the pandemic may have affected the participants’ experiences of taking part in the VRAPT intervention. In the data, it is clear that participants in general felt confident that the clinic took the necessary precautions to prevent them from becoming infected. For some participants, Covid-19 did not cause any worry.

*“It wasn’t a big deal, I don’t see what the problem would be during a pandemic … Nothing changed. It didn’t affect anything.” (P3)*

*“Well, what we did was to keep our distance during sessions and have good hand hygiene and that was what mattered in those moments. Then you could see that there were corona routines with distance and hand sanitizers … The mask you wore, before you put on the VR glasses, that probably reduced the risk of getting infected.” (P4)*

## 4 Discussion

The aim of this study was to describe forensic psychiatric patients’ experiences of having undergone the newly revised VRAPT intervention at a high-security forensic psychiatric clinic. The participants had very diverse experiences, and important findings not only specifically related to the VRAPT intervention itself but to the forensic psychiatric care setting emerged. Some participants had distinctly positive experiences, where specifically skills training through role-plays in VR was emphasized as something beneficial that possibly could be extended. Challenges in the form of lack of motivation for aggression treatment among participants, and with technological limitations were described. In total, five content categories were manifest in the data: 1. Therapeutic process, 2. VRAPT method, 3. VR technology, 4. Previous treatment experiences, 5. Challenges to treatment of aggression, and 6. Unexpected experiences.

### 4.1 Therapeutic process

The participants described that the relationship with their therapists was an important aspect of their VRAPT treatment. Most participants described the professional relationship in a positive way, highlighting aspects such as open communication and feeling safe during the treatment. This can be related to what in psychological treatment literature is referred to as therapeutic, or working alliance (64, 65). For example, having the therapist describe clearly what was going to happen in a role-play could lead to increased feelings of safety, even in situations that could be challenging for the participant. However, there were also participants who described feelings of sadness and stigmatization, giving voice to feelings resembling hopelessness (e.g., “*I am also a human, I have made a mistake and am here to pay for it, doing all the programs, treatments, attending meetings and there is nothing more*.”). Given the context of the study, forensic psychiatry, possible impact of multiple stigmas identity (e.g., severe mental disorder, criminal history, ethnic/racial minorities) and self-stigma (66) may have affected the participants’ experiences, especially regarding the therapeutic process in the study. We believe it is important to highlight here the importance of a critical, reflective and ethical perspective in the treatment of aggression with forensic patients, as we consider that the treatment of aggression (and in general, any treatment of vulnerable groups) cannot be based exclusively on following a manual. Maintaining a global perspective of the patient, and a holistic vision in relation to the treatment, is fundamental in forensic settings. Also, working with and following up the patient’s experiences from the therapeutic collaboration is important, regardless of whether it is specified in a manual, something which may facilitate to discover and manage when patients have negative experiences during the treatment. In settings such as the one studied here, misunderstandings may arise between therapist and patient and the coercive nature of the care in general may affect the patient’s overall experiences, which makes focused work with the therapeutic collaboration crucial. A possible paternalistic stance in the therapeutic collaboration might be seen in some quotes; *“My therapist had, I think, a genuine sense of developing the patient in the flaws that they possess.”* Here, the context of the study must be considered, as high-security forensic psychiatry is a treatment context with clear coercive elements where such attitudes might occur and affect the care.

In terms of agreement on treatment goals, an aspect commonly described as a crucial part of the working alliance (64, 65), several participants found it difficult to recall the specific treatment goals. This can be related to several aspects, e.g. the fact that the interviews were conducted 12 weeks post treatment termination, possibly affecting their memory of specific treatment goals. Other aspects possibly influencing this could be the complex clinical profile of forensic psychiatric patients, with multiple difficulties including severe mental disorders (e.g., psychosis) and cognitive deficits (67, 68). In that regard, it is even more striking that one of the participants was able to elaborate on and relate the treatment goals to his own development. Interestingly, one of the subcategories that emerged, which is closely related to treatment goals, was matching of treatment to patient’s needs. It seems as if some of the participants could not agree with the therapist on the overarching goal of the treatment — managing the participant’s own aggression — since that would demand too much of the participant. Differences in preconceptions between participants and therapists on the nature of VR-assisted treatment of aggression, affecting the participant’s perceived value of the treatment, were evident. Not surprisingly, spending initial time on a common understanding of goal and case formulation seems crucial for the participant’s experience of the therapeutic process (69).

One part of the therapeutic process, which was highlighted as beneficial by several participants, was the skills training in the virtual worlds. During VR-assisted role-plays, some participants described discomfort due to them practicing managing challenging situations, i.e. it was difficult to find strategies for coping with social situations in which they were confronted with situations that had provoked aggression in the past. For some, this feeling of discomfort was the source of motivation to cope and learn new communication and self-regulation strategies. Continued skills training and practicing communication strategies were highlighted as remaining treatment needs, something which should be considered in future revisions of the VRAPT intervention.

### 4.2 VRAPT method

The majority of participants, albeit some with initial ambivalence, were satisfied with the outcomes of their VRAPT treatment, emphasizing the importance of talking about aggression (compared to previous experiences of not being allowed to discuss the subject), learning to recognize and interpret facial expressions and social situations, learning and practicing communication skills, and rehearse management of stressful situations through VR role-plays (14, 31). However, several participants mentioned homework assignments as demotivating, being too repetitive and reminding them of school experiences, while some participants described them as a central part of the treatment. Notably, despite staff being technically available 24/7, a lack of staff support with the homework assignments was mentioned. Homework assignments play an important role in cognitive and cognitive-behavioral psychological treatments, emphasized early by Beck and colleagues (70), in practicing and reinforcing the skills learned during treatment in the patient’s everyday life (71). Here, future revisions of the VRAPT intervention need to assimilate these experiences into a renewed take on homework assignments, reducing repetitive wording and possibly directly involving ward staff in homework assignments to increase the likelihood of completing them successfully. Hopefully, this may aid transference of learned skills to patients’ everyday lives but is still to be evaluated in coming studies. Another critical variable to improve the impact of this kind of therapeutic intervention is the commitment of the patient or user (72), which should be measured in coming studies.

During interviews, deviations from the VRAPT methodology, e.g., performing face-to-face role-plays instead of VR-assisted role-plays and altering the direction of the treatment, were described. The provided descriptions suggest that these may have been necessary alterations from a clinical point of view. However, this obviously affects VRAPT treatment integrity. In larger, quantitative evaluations of the VRAPT intervention, the limit to which treatment integrity must be maintained in order for the treatment to be conceptualized as “VRAPT” must be clarified. In clinical everyday practice this may, however, prove challenging. Forensic psychiatric care has many facets, and in the course of a manualized treatment (e.g., VRAPT) an indeterminate number of events occur, which implies incorporating different institutional levels when implementing and evaluating new virtual reality treatments (73, 74).

### 4.3 VR technology

The majority of participants had positive experiences of using the VR technology and perceived it as “novel” and “fun”, something which in itself may have been a motivating factor for participating in the treatment. Participants formulated areas within forensic psychiatry, specifically related to different kinds of skills training, where they saw VR as having potential for improving interventions. The current results are believed to add to previous descriptions of VR as a way of increasing the possibilities for bridging the “gap” between the real world and the therapeutic setting and as motivation-enhancing within forensic settings (46).

Considering main concepts in VR — presence, immersion, and embodiment (17–20) — our results suggest that the applied technology created a sense of presence and immersion for many participants. In some cases, participants described that the technology was good enough for them “to be there” (i.e., experience presence). However, some participants reported not feeling immersed enough, and attributed this to technological limitations e.g. graphics. Other technological limitations described by participants were related to a sense of embodiment, in that the participants found the lack of being able to perform their own physical movements in the VR world limiting (e.g., not being able to back away during a discussion). However, no severe or persisting cases of cybersickness were reported, with such experiences limited to the beginning of VRAPT. In previous studies on forensic psychiatric patients, cybersickness has been one of the causes of dropout (41). Overall, the technology applied in the current VRAPT intervention seems valid for a “good enough” experience without major side-effects for the majority of participants. However, the descriptions of technological limitations hindering a sense of immersion and embodiment, can be considered in future revisions of the software.

### 4.4 Previous treatment experiences

None of the participants described having previously received any type of treatment focused specifically on aggression problems, making comparisons of the VRAPT intervention to other aggression treatments impossible. A positive approach towards interventions such as VRAPT was noted. However, participants spontaneously shared their general experiences of forensic psychiatric care. Many of these were negative experiences of being locked up and frustration concerning the forensic psychiatric care system. For the patients, adapting to the forensic institution may often be a challenging process, and focusing on development of a sense of self and connectedness has been suggested to help enhance recovery (75).

In order to understand and treat aggression in a forensic psychiatric context, it is necessary to include those situations that incite patients to aggression within the institution. Previous studies have revealed that both social climate and sense of community predict aggressiveness in high-security hospitals (76). Other studies (77) have indicated that institutional restrictions and patients’ psychopathology influence treatment course and outcome of forensic psychiatric patients. With this in mind, all treatments within the forensic psychiatric setting will undoubtedly be influenced by the patients’ previous experiences from this, or similar, settings. This is a more general note than something specific to the VRAPT intervention, however.

### 4.5 Challenges to treatment of aggression

Some of the participants described their own attitudes associated with instrumental (or “recreational”) violence, where, for some, the desire to harm was a fundamental part of how they identified themselves. Descriptions of thought-out manipulations of the therapeutic process may be in line with a more instrumental aggression, and with psychopathy in general, but it is unclear how an assessment of psychopathy could uniquely inform treatment and rehabilitation strategies (78, 79). VRAPT was not designed as a treatment for instrumental aggression, which may be why these participants in previous content categories provided negative experiences of the intervention. The established categorization of aggression into reactive, instrumental and psychotic aggression has been confirmed in long-stay public psychiatric hospitals (80), and we consider this categorization to be possibly useful for tailoring treatment for aggression in forensic settings. In its current form, VRAPT should be specifically directed for persons with predominantly reactive aggression. However, aggression should not be understood as a linear and categorizable process as it is more adequately described as complex and multidimensional but may be communicated more easily in categories. Thus, the multifaceted nature of dysfunctional aggression must be acknowledged in interventions, while interventions such as VRAPT might need to focus on the type of aggression where a potential benefit for the participants (and the safety of society) is most obvious – in the case of VRAPT the reactive aggression. On the other hand, we believe that it is important to highlight responsivity as modeled in the Risk-Need-Responsivity (RNR) model (81) during the VR-assisted aggression treatment because of the complexity of forensic psychiatric patients, and as well as the challenges therapists must consider in clinical relationships with forensic clients (82).

### 4.6 Unexpected experiences

As described previously, the VRAPT intervention that the participants in this study completed was initiated at the same time as Covid-19 started to spread over the world. However, when asked about in the interviews, this was described as being handled with specific routines and not creating any feelings of being unsafe among the participants. This suggests that the routines related to Covid-19 were assimilated as part of the everyday care at the clinic. During the pandemic, many changes occurred in forensic psychiatric institutions (83). However, in this study, the general perception of the participants was that the clinic handled it adequately and that they felt confident in the actions taken. In summary, the participants described that the pandemic did not negatively influence their participation in the VRAPT treatment.

### 4.7 Limitations and strengths

For the research team, the entire logistical organization of the study, including the interviews, was difficult and complex, as the study was initiated at the same time that the COVID-19 pandemic incapacitated the world. Nevertheless, the patients do not report any limitations due to COVID-19 affecting their participation in a negative way. Another possible limitation was that the interviews first were conducted 12 weeks after the VRAPT intervention was completed, to not affect a quantitative follow-up that was conducted 12 weeks post-VRAPT. It may have been that conducting interviews with patients just after their completion of VRAPT could have resulted in a somewhat different description of their experiences. Given the scarcity of qualitative studies in this field, such limitations cannot be completely avoided and must be recognized. Another limitation is something which must be handled in qualitative studies in general – sometimes participants’ quotes may seem to apply to different categories. For example, the subcategories “treatment outcomes” and “remaining treatment needs” in this study do show similarities. However, in the interpretive process, the proposed content categories and subcategories were deemed to be sufficiently distinct. Furthermore, the sample only consisted of seven participants, something which could be considered as a smaller sample even in qualitative research (60). However, the study was performed with a narrow focus and on a total sample from a clinical pilot study, and our assessment was that data saturation was achieved through the interviews. The inherent bias of the interviewer and researchers who conducted the analyses, all forensic psychologists with considerable experience from working in forensic psychiatry, during the study and interpretation of the data should also be considered a limitation. A final limitation of this study was the imbalance in the gender of the participants — 6 men and 1 woman. This makes it difficult to have a broad perspective of how female patients perceive undergoing the VRAPT intervention. However, this gender imbalance is in line with the gender distribution of forensic psychiatric patients in general in Sweden. In the future, it is recommended that VR-assisted treatment of aggression should involve a larger proportion of, or focus exclusively on, female participants.

A significant strength of this study was that all participants who completed VRAPT at the time of the study agreed to participate in interviews. Notably, participants described that they were proud to participate and to be part of the technological development in society. Considerations on credibility, dependability and transferability to ensure trustworthiness of outcomes (61) were made through assuring diversity in the sample with various cultural backgrounds, ages and levels of education, various psychiatric problems, different types of crimes and varying length of stay in forensic psychiatry.

## 5 Conclusion

This study reports forensic psychiatric patients’ experiences of recently having undergone VR-assisted aggression treatment through the VRAPT intervention. Interviews demonstrated skills training through role-plays in VR as something that was perceived as beneficial by several participants and suggested that this part could be extended. Challenges in the form of lack of motivation for aggression treatment among participants, and with technological limitations were described. The categories that emerged bottom-up from interviews should be put in relation to clinical challenges within the specific context; patients’ experiences from the intervention need to be understood in relation to how psychotic functioning, including paranoia, may affect their reaction to VR-assisted interventions and to aggression management in particular. Nevertheless, previous studies indicate that VR-assisted interventions, also those focusing on aggression, are feasible in these clinical settings (14, 41, 42).

The study highlights the importance of educating providers (in this case therapists) on how to use VR, its possibilities and technological limitations. Continued qualitative studies on patients’ experiences is recommended, given that this can help care providers tailor treatment interventions to patients’ needs.

## Data availability statement

The raw data supporting the conclusions of this article will be made available by the authors, without undue reservation.

## Ethics statement

The studies involving humans were approved by Swedish Ethical Review Authority (Dnr: 2019-02337; 2020-06317). The studies were conducted in accordance with the local legislation and institutional requirements. The participants provided their written informed consent to participate in this study.

## Author contributions

FG: Conceptualization, Data curation, Funding acquisition, Investigation, Methodology, Project administration, Writing – original draft, Writing – review & editing, Formal analysis. PE: Conceptualization, Methodology, Supervision, Writing – review & editing. SP: Conceptualization, Supervision, Writing – review & editing. KS: Conceptualization, Supervision, Writing – review & editing. WV: Conceptualization, Writing – review & editing. MW: Conceptualization, Formal analysis, Funding acquisition, Methodology, Project administration, Supervision, Writing – review & editing.

## References

[1] Munthe C Radovic S Anckarsäter H. Ethical issues in forensic psychiatric research on mentally disordered offenders. Bioethics. (2010) 24:35–44. doi: 10.1111/j.1467-8519.2009.01773.x 20017746

[2] Shapiro DL. Ethical issues in forensic psychology and psychiatry. Ethics Med Public Health. (2016) 2:45–58. doi: 10.1016/j.jemep.2016.01.015

[3] Völlm B Bartlett P McDonald R. Ethical issues of long-term forensic psychiatric care. Ethics Med Public Health. (2016) 2:36–44. doi: 10.1016/j.jemep.2016.01.005

[4] Bogaerts S Polak M Spreen M Zwets A. High and low aggressive narcissism and anti-social lifestyle in relationship to impulsivity, hostility, and empathy in a group of forensic patients in the Netherlands. J Forensic Psychol Pract. (2012) 12:147–62. doi: 10.1080/15228932.2012.650144

[5] Lobbestael J Cima M Lemmens A. The relationship between personality disorder traits and reactive versus proactive motivation for aggression. Psychiatry Res. (2015) 229:155–60. doi: 10.1016/j.psychres.2015.07.052 26213380

[6] Krona H Anckarsäter H Nilsson T Hofvander B. Patterns of lifetime criminality in mentally disordered offenders–findings from a nationally representative cohort. Front Psychiatry. (2021) 12. doi: 10.3389/fpsyt.2021.564171 PMC8357977 34393835

[7] Gatner DT Moulden HM Mamak M Chaimowitz GA. At risk of what? Understanding forensic psychiatric inpatient aggression through a violence risk scenario planning lens. Int J Forensic Ment Health. (2021) 20:398–407. doi: 10.1080/14999013.2021.1899343

[8] Wallinius M. Aggressive antisocial behavior-clinical, cognitive, and behavioral covariates of its persistence. Lund: Doctoral dissertation, Lund University (2012).

[9] González Moraga FR Tuente S Perrin S Enebrink P Sygel K Veling W. New developments in virtual reality-assisted treatment of aggression in forensic settings: the case of VRAPT. Front Virtual Real. (2022) 2: doi: 10.3389/frvir.2021.675004

[10] González Moraga FR Garcia D Billstedt E Wallinius M. Facets of psychopathy, intelligence and aggressive antisocial behaviors in young violent offenders. Front Psychol. (2019) 10:984. doi: 10.3389/fpsyg.2019.00984 31139107 PMC6527586

[11] Howner K Andiné P Bertilsson G Hultcrantz M Lindström E Mowafi F. Mapping systematic reviews on forensic psychiatric care: a systematic review identifying knowledge gaps. Front Psychiatry. (2018) 9:452. doi: 10.3389/fpsyt.2018.00452 30319459 PMC6167556

[12] Lee AH DiGiuseppe R. Anger and aggression treatments: a review of meta-analyses. Curr Opin Psychol. (2018) 19:65–74. doi: 10.1016/j.copsyc.2017.04.004 29279226

[13] Trestman RL. Treating aggression in forensic psychiatric settings. J Am Acad Psychiatry Law. (2017) 45:40–3. 28270461

[14] Sygel K Wallinius M. Immersive virtual reality simulation in forensic psychiatry and adjacent clinical fields: a review of current assessment and treatment methods for practitioners. Front Psychiatry. (2021) 12. doi: 10.3389/fpsyt.2021.673089 PMC8193033 34122189

[15] Geraets CN van der Stouwe EC Pot-Kolder R Veling W. Advances in immersive virtual reality interventions for mental disorders: A new reality? Curr Opin Psychol. (2021) 41:40–5. doi: 10.1016/j.copsyc.2021.02.004 33714892

[16] Geraets C Wallinius M Sygel K. Use of virtual reality in psychiatric diagnostic assessments: A systematic review. Front Psychiatry. (2022) 260. doi: 10.3389/fpsyt.2022.828410 PMC8918631 35295778

[17] Slater M. Place Illusion and Plausibility can lead to realistic behaviour in immersive virtual environments. Philos Trans R Soc London. (2009) 364:74. doi: 10.3389/frobt.2016.00074 PMC2781884 19884149

[18] Bailey JO Bailenson JN Casasanto D. When does virtual embodiment change our minds? Presence. (2016) 25:222–33. doi: 10.1162/PRES_a_00263

[19] Riva G. Virtual reality in clinical psychology. In reference module in neuroscience and biobehavioral psychology. Amsterdam: Elsevier (2020).

[20] Slater M. Immersion and the illusion of presence in virtual reality. Br J Psychol. (2018) 109:431–3. doi: 10.1111/bjop.12305 29781508

[21] Lanier J. Dawn of the new everything: A journey through virtual reality. London: Random House (2017).

[22] Cummings JJ Bailenson JN. How immersive is enough? A meta-analysis of the effect of immersive technology on user presence. Media Psychol. (2016) 19:272–309. doi: 10.1080/15213269.2015.1015740

[23] Lee KM. Presence, explicated. Communication Theory. (2004) 14:27–50. doi: 10.1093/ct/14.1.27

[24] Ticknor B. Virtual reality and the criminal justice system: exploring the possibilities for correctional rehabilitation. Lanham: Lexington Books (2018).

[25] Slater M Spanlang B Corominas D. Simulating virtual environments within virtual environments as the basis for a psychophysics of presence. ACM Trans Graphics (TOG). (2010) 29:1–9. doi: 10.1145/1778765.1778829

[26] Kilteni K Groten R Slater M. The sense of embodiment in virtual reality. Presence: Teleoperators Virtual Environments. (2012) 21:373–87. doi: 10.1162/PRES_a_00124

[27] Fromberger P Jordan K Müller JL. Use of virtual reality in forensic psychiatry: a new paradigm? Der Nervenarzt. (2014) 85:298–303. doi: 10.1007/s00115-013-3904-7 24549691

[28] Kip H Bouman YHA Kelders SM van Gemert-Pijnen L. J. E. W. C. eHealth in treatment of offenders in forensic mental health: a review of the current state. Front Psychiatry. (2018) 9:42. doi: 10.3389/fpsyt.2018.00042 29515468 PMC5826338

[29] Kisker J Gruber T Schöne B. Experiences in VR entail different processes of retrieval as opposed to conventional laboratory settings: A study on human memory. Curr Psychol. (2019) 40:3190–7. doi: 10.1007/s12144-019-00257-2

[30] Spiegel B. VRx: how virtual therapeutics will revolutionize medicine. New York: Basic Books (2020).

[31] Tereso IM Ramos ALG Santos BR Costa JPM. Virtual reality and forensic mental health. In: In digital therapies in psychosocial rehabilitation and mental health. IGI Global (2022). p. 245–60. doi: 10.4018/978-1-7998-8634-1

[32] Ireland JL. Individual assessments of aggression: Accounting for core factors. In: Ireland JL Ireland CA Birch P, editors. Violent and sexual offenders: Assessment, treatment and management, 2nd ed. Routledge, Abingdon (2018). p. 203–27.

[33] Lewis M Ireland JL. Understanding motives for aggression in forensic psychiatric patients: A preliminary study. J Forensic Psychiatry Psychol. (2019) 30:496–509. doi: 10.1080/14789949.2019.1570541

[34] Bjørnebekk G Howard R. Validation of a motivation-based typology of angry aggression among antisocial youths in Norway. Behav Sci Law. (2012) 30:167–80. doi: 10.1002/bsl.2007 22388964

[35] Howard R Howells K Jinks M McMurran M. A quadripartite typology of violence (QTV): Relationships with functions of aggression in violent youths. In: Needham I Callaghan P Palmstierna T Nijman H Oud N, editors. Violence in clinical psychiatry. Kavanah, The Netherlands (2009). p. 342–5.

[36] Camp JP Skeem JL Barchard K Lilienfeld SO Poythress NG. Psychopathic predators? Getting specific about the relation between psychopathy and violence. J consulting Clin Psychol. (2013) 81:467. doi: 10.1037/a0031349 PMC3978196 23316742

[37] Daffern M Howells K Ogloff J. What’s the point? Towards a methodology for assessing the function of psychiatric inpatient aggression. Behav Res Ther. (2007) 45:101–11. doi: 10.1016/j.brat.2006.01.011 16530162

[38] Fletcher A Crowe M Manuel J Foulds J. Comparison of patients’ and staff’s perspectives on the causes of violence and aggression in psychiatric inpatient settings: An integrative review. J Psychiatr Ment Health Nurs. (2021) 28:924–39. doi: 10.1111/jpm.12758 33837640

[39] Smeijers D Koole SL. Testing the effects of a virtual reality game for aggressive impulse management (VR-GAIME): study protocol. Front Psychiatry. (2019) 10:83. doi: 10.3389/fpsyt.2019.00083 30863328 PMC6399131

[40] Tuente S Bogaerts S Van Ijzendoorn S Veling W. Effect of virtual reality aggression prevention training for forensic psychiatric patients (VRAPT): study protocol of a multi-center RCT. BMC Psychiatry. (2018) 18:1–9. doi: 10.1186/s12888-018-1830-8 30081863 PMC6091200

[41] Smeijers D Bulten EH Verkes RJ Koole SL. Testing the effects of a virtual reality game for aggressive impulse management: A preliminary randomized controlled trial among forensic psychiatric outpatients. Brain Sci. (2021) 11:1484. doi: 10.3390/brainsci11111484 34827483 PMC8615718

[42] Tuente S Bogaerts S Bulten E Keulen-de Vos M Vos M Bokern H. Virtual reality aggression prevention therapy (VRAPT) versus waiting list control for forensic psychiatric inpatients: a multicenter randomized controlled trial. J Clin Med. (2020) 9:2258. doi: 10.3390/jcm9072258 32708637 PMC7409015

[43] Lobbestael J Cima MJ. Virtual reality for aggression assessment: The development and preliminary results of two virtual reality tasks to assess reactive and proactive aggression in males. Brain Sci. (2021) 11:1653. doi: 10.3390/brainsci11121653 34942955 PMC8699434

[44] Quarmley M Vafiadis A Jarcho JM. Irritability and rejection-elicited aggression in adolescents and young adults. J Child Psychol Psychiatry. (2023) 64.9. doi: 10.1111/jcpp.13804 37036378

[45] Bailenson J. Experience on demand: What virtual reality is, how it works, and what it can do. New York: WW Norton & Company (2018).

[46] Kip H Kelders SM Weerink K Kuiper A Brüninghoff I Bouman YH. Identifying the added value of virtual reality for treatment in forensic mental health: a scenario-based, qualitative approach. Front Psychol. (2019) 10:406. doi: 10.3389/fpsyg.2019.00406 30873093 PMC6400887

[47] Lutz M Zani D Fritz M Dudeck M Franke I. A review and comparative analysis of the risk-needs-responsivity, good lives, and recovery models in forensic psychiatric treatment. Front Psychiatry. (2022) 13:988905. doi: 10.3389/fpsyt.2022.988905 36386990 PMC9659584

[48] Söderberg A Wallinius M Munthe C Rask M Hörberg U. Patients’ experiences of participation in high-security, forensic psychiatric care. Issues Ment Health Nurs. (2022) 43:683–92. doi: 10.1080/01612840.2022.2033894 35130107

[49] Ivarsson D Delfin C Enebrink P Wallinius M. Pinpointing change in virtual reality assisted treatment for violent offenders: a pilot study of Virtual Reality Aggression Prevention Training (VRAPT). Frontiers in psychiatry. (2023) 14, 1239066. doi: 10.3389/fpsyt.2023.1239066 38034926 PMC10687219

[50] Anderson CA Bushman BJ. Human aggression. Psychology. (2002) 53:27. doi: 10.1146/annurev.psych.53.100901.135231 11752478

[51] Anderson CA Carnagey NL. Violent evil and the general aggression model. In: The social psychology of good and evil (2004) New York: Guilford. p. 168–92.

[52] DeWall CN Anderson CA Bushman BJ. The general aggression model: Theoretical extensions to violence. Psychol violence. (2011) 1:245. doi: 10.1037/a0023842

[53] Kyngäs H. Inductive content analysis. In: In The application of content analysis in nursing science research. Springer, Cham (2020). p. 13–21.

[54] Vears DF Gillam L. Inductive content analysis: A guide for beginning qualitative researchers. Focus Health Prof Education: A Multi-disciplinary J. (2022) 23:111–27. doi: 10.11157/fohpe.v23i1.544

[55] Fogg BJ. Persuasive technology: using computers to change what we think and do Vol. 2002. Ubiquity (2002) Burlington, MA: Morgan Kaufmann. p. 2.

[56] Blitz MJ. Extended reality, mental liberty, and state power in forensic settings. AJOB Neurosci. (2022) 13:173–6. doi: 10.1080/21507740.2022.2086647 35797123

[57] Kellmeyer P Biller-Andorno N Meynen G. Ethical tensions of virtual reality treatment in vulnerable patients. Nat Med. (2019) 25:1185–8. doi: 10.1038/s41591-019-0543-y 31359003

[58] Ligthart S Meynen G Biller-Andorno N Kooijmans T Kellmeyer P. Is virtually everything possible? The relevance of ethics and human rights for introducing extended reality in forensic psychiatry. AJOB Neurosci. (2022) 13:144–57. doi: 10.1080/21507740.2021.1898489 33780323

[59] Lidz CW Appelbaum PS. The therapeutic misconception: problems and solutions. Med Care. (2002) 40(9):V55–63. doi: 10.1097/00005650-200209001-00008 12226586

[60] Hennink M Kaiser BN. Sample sizes for saturation in qualitative research: A systematic review of empirical tests. Soc Sci Med. (2022) 292:114523. doi: 10.1016/j.socscimed.2021.114523 34785096

[61] Graneheim UH Lundman B. Qualitative content analysis in nursing research: concepts, procedures and measures to achieve trustworthiness. Nurse Educ Today. (2004) 24:105–12. doi: 10.1016/j.nedt.2003.10.001 14769454

[62] Bengtsson M. How to plan and perform a qualitative study using content analysis. NursingPlus Open. (2016) 2:8–14. doi: 10.1016/j.npls.2016.01.001

[63] Graneheim UH Lindgren BM Lundman B. Methodological challenges in qualitative content analysis: A discussion paper. Nurse Educ Today. (2017) 56:29–34. doi: 10.1016/j.nedt.2017.06.002 28651100

[64] Bordin ES. “Theory and research on the therapeutic working alliance: New directions,” in The Working Alliance: Theory, Research, and Practice, eds Horvath A. O. Greenberg L. S. Hoboken, NJ: John Wiley and Sons (1994). p. 13–37.

[65] Clercx M de Vogel V Lancel M Keulen-de Vos M. The influence of therapy alliance and treatment motivation in patients with Cluster B personality disorders on incidents in forensic hospitals. J Forensic Pract. (2021) 23:272–84. doi: 10.1108/JFP-05-2021-0022

[66] West ML Yanos PT Mulay AL. Triple stigma of forensic psychiatric patients: Mental illness, race, and criminal history. Int J Forensic Ment Health. (2014) 13:75–90. doi: 10.1080/14999013.2014.885471

[67] Andiné P Bergman H. Focus on brain health to improve care, treatment, and rehabilitation in forensic psychiatry. Front Psychiatry. (2019) 10:840. doi: 10.3389/fpsyt.2019.00840 31849721 PMC6901922

[68] Laporte N Ozolins A Westling S Westrin Å Wallinius M. Clinical characteristics and self-harm in forensic psychiatric patients. Frontiers in psychiatry. (2021) 12, 698372. doi: 10.3389/fpsyt.2021.698372 34408680 PMC8365140

[69] Hart S Sturmey P Logan C McMurran M. Forensic case formulation. Int J Forensic Ment Health. (2011) 10:118–26. doi: 10.1080/14999013.2011.577137

[70] Beck AT. Cognitive therapy of depression. New York: Guilford press (1979). 10.1046/j.1440-1614.2002.t01-5-01015.x 11982561

[71] Tang W Kreindler D. Supporting homework compliance in cognitive behavioural therapy: essential features of mobile apps. JMIR Ment Health. (2017) 4:e5283. doi: 10.2196/mental.5283 PMC5481663 28596145

[72] Fleming TM De Beurs D Khazaal Y Gaggioli A Riva G Botella C. Maximizing the impact of e-therapy and serious gaming: time for a paradigm shift. Front Psychiatry. (2016) 7:65. doi: 10.3389/fpsyt.2016.00065 27148094 PMC4834305

[73] Papalia N Spivak B Daffern M Ogloff JR. A meta-analytic review of the efficacy of psychological treatments for violent offenders in correctional and forensic mental health settings. Clin Psychology: Sci Pract. (2019) 26:e12282. doi: 10.1111/cpsp.12282

[74] Kouijzer MM Kip H Bouman YH Kelders SM. Implementation of virtual reality in healthcare: a scoping review on the implementation process of virtual reality in various healthcare settings. Implementation Sci Commun. (2023) 4:1–29. doi: 10.1186/s43058-023-00442-2 PMC10276472 37328858

[75] Clarke C Lumbard D Sambrook S Kerr K. What does recovery mean to a forensic mental health patient? A systematic review and narrative synthesis of the qualitative literature. J Forensic Psychiatry Psychol. (2016) 27:38–54. doi: 10.1080/14789949.2015.1102311

[76] Puzzo I Aldridge-Waddon L Bush E Farr C. The relationship between ward social climate, ward sense of community, and incidents of disruptive behavior: a study of a high secure psychiatric sample. Int J Forensic Ment Health. (2019) 18:153–63. doi: 10.1080/14999013.2018.1532972

[77] Franke I Büsselmann M Streb J Dudeck M. Perceived institutional restraint is associated with psychological distress in forensic psychiatric inpatients. Front Psychiatry. (2019) 10:410. doi: 10.3389/fpsyt.2019.00410 31244698 PMC6580144

[78] Salekin RT. Psychopathy and therapeutic pessimism: Clinical lore or clinical reality? (2002) ) 22.1:79–112. Clinical. doi: 10.1016/S0272-7358(01)00083-6 11793579

[79] Larsen RR Jalava J Griffiths S. Are Psychopathy Checklist (PCL) psychopaths dangerous, untreatable, and without conscience? A systematic review of the empirical evidence. Psychology Public Policy Law. (2020) 26:297. doi: 10.1037/law0000239

[80] Quanbeck CD McDermott BE Lam J Eisenstark H Sokolov G Scott CL. Categorization of aggressive acts committed by chronically assaultive state hospital patients. Psychiatr Serv. (2007) 58:521–8. doi: 10.1176/appi.ps.58.4.521 17412855

[81] Skeem JL Steadman HJ Manchak SM. Applicability of the risk-need-responsivity model to persons with mental illness involved in the criminal justice system. Psychiatr Serv. (2015) 66:916–22. doi: 10.1176/appi.ps.201400448 25930045

[82] Chudzik L Aschieri F. Clinical relationships with forensic clients: A three-dimensional model. Aggression Violent Behav. (2013) 18:722–31. doi: 10.1016/j.avb.2013.07.027

[83] Terkildsen MD Vestergaard LK Møllerhøj J Sørensen LU. Forensic psychiatric patients’ Perspectives on COVID-19 prevention measures: A qualitative study. J Forensic Psychol Res Pract. (2022) 24.2: 245–67. doi: 10.1080/24732850.2022.2118095

